# The early‐life stress induced by oxytocin inhibition in p53 knockout mouse dams increases adulthood tumorigenesis in first and second generations

**DOI:** 10.1002/cnr2.1625

**Published:** 2022-05-11

**Authors:** Massimo Stendardo, Chiara Renzi, Rani Pallavi, Niccolò Roda, Valentina Gambino, Francesca Casciaro, Giuseppe Persico, Marco Giorgio

**Affiliations:** ^1^ Experimental Oncology Department IRCCS‐European Institute of Oncology Milan Italy; ^2^ University of Modena and Reggio Emilia Modena Italy; ^3^ Department of Biomedical Sciences University of Padua Padova Italy

**Keywords:** cancer risk factor, early‐life stress, oxytocin, p53 KO mice, parental care

## Abstract

**Background:**

Early‐life stress due to poor parental care has been suggested to increase cancer risk, though, so far, no experimental evidence established a link between defective parental behavior and spontaneous tumorigenesis in progeny. Essential maternal behavior is regulated, in particular, by the oxytocin (OT) hormonal circuit, which in turn responds to stimuli from the offspring and impinges on the central nervous systems.

**Methods:**

By providing L‐368,899 OT receptor (OTR) inhibitor to lactating mothers, we set up a model of defective maternal care in p53 knockout mice.

**Results:**

The progeny of these dams showed, later in life, higher cortisol levels, shortened life span and increased tumorigenic potential of bone marrow cells (BMC). Notably, these phenotypes were transmitted to the following generation.

**Conclusions:**

Therefore, the inhibition of OT function in mothers is a novel paradigm of early‐life stress that is inherited across generations and increases cancer risk in tumor‐prone mice.

## 1 INTRODUCTION

Receiving inadequate parental care, as other psychosocial stresses, represents an emerging risk factor for cancer development.^1^, ^2^ Despite of several epidemiological evidences for adverse childhood experiences as a cancer risk factor,^3^ effect of parental care, a key childhood psychosocial exposure, on the risk of cancer at older age is not well studied. There are only two studies in humans that addressed the effect of parental care and revealed that poor parental care is associated with increased incidence of all‐site cancer risk^4^ and worse breast cancer prognosis.^5^ Mouse models with the possibilities of higher level of environmental control, exploration of intervention and genetic manipulations offer a unique opportunity to understand the detrimental effect of early life experiences. Studies in mice using mother‐pup separation after birth showed that the puppies during adult‐hood are more sensitive to chemically induced breast carcinogenesis in BALB/c mice^6^ and in Wistar rats^7^ and showed increased progression of melanoma and breast cancer xenografts in C57BL/6 mice^8^ or Fischer 344 rats.^9^ These studies are informative but fail to represent the natural process of tumor development and progression. In this regard, studies addressing the effect of insufficient maternal care effect on cancer development in offspring using spontaneous tumor mouse model is needed. Further, maternal separation, manipulation procedure used in these studies seems to have several methodological pitfalls that could influence the reliability of its results.

The quality of parental care is determined by several environmental and organismal components,^10^, ^11^ among which the maternal bond ruled by oxytocin (OT) plays a crucial role.^12^

OT is a vertebrate nonapeptide hormone, highly conserved across all mammals, which plays a critical role in several physiological processes^13^ related to reproductive and social behavior. OT is mostly synthesized in the supraoptic and paraventricular nuclei of the hypothalamus and released in the posterior part of the pituitary gland. Minor quantities of OT are secreted by gonads, prostate,^14^ and colon.^15^ The receptor for OT (OTR) is a *G*_q_ protein‐coupled receptor and it is expressed almost ubiquitously, being absent only in hepatocytes and lung cells. OTR generates inositol trisphosphate and diacylglycerol through the activation of phospholipase C, thereby fostering Ca^2+^ signaling and mitogen‐activated protein kinase pathway within the cell.^16^ Activation of reward circuits induced by OT in the mother reinforces the attachment to the newborn and contributes to develop helpful interactions with the litters.^17^ In rodents, OT plays a fundamental role in establishing the bond between the mother and the puppy, which is in turn crucial for the execution of effective parental care.^18^ In fact, the genetic or pharmacological inhibition of OTR counteracts the maternal behavior in laboratory^19^, ^20^ or prairie voles^21^ and triggers in the offspring the stress‐induced activation of the hypothalamic–pituitary–adrenal axis (HPA).^20^

In this study, we addressed the role of deviant maternal behavior in the induction of spontaneous tumorigenesis in the progeny in a preclinical setting. We have used p53 KO mice as these mice develop normally but are prone to the spontaneous development of tumor by 6 months of age. Thus, making this mouse suitable to study spontaneous tumorigenesis. To this aim, we inhibited oxytocin signaling by using the L‐368,899 OTR antagonist,^22^ and investigated the consequent effects of altered mother‐newborn relationship on survival, tumor incidence and spectrum of cancer in the p53 deleted tumor‐prone mouse model.^23^

## 2 MATERIALS AND METHODS

### 2.1 Mice breeding, genotyping, and treatment

C57Bl/6 p53 knockout mice were originally obtained from Jackson laboratories (B6.129S2‐Trp53tm1Tyj/J). Wild type C57Bl/6 were purchased from Charles River Laboratories, Calco, Italy. Breeding and experiments were carried out on mice maintained in a specific‐pathogen free (FELASA) temperature‐controlled room (temperature 21 ± 1°C, relative humidity 60 ± 10%), single‐cage housed for the life span determination, at IEO (Ministry of Health authorization: DM N°86/2005–17/06/2005) with a 12 h light/12 h dark cycle (7:00–19:00) and food (Standard 2018S Teklad Global, 18% protein, 3.3 kcal/g, rodent diet by Harlan Teklad, Madison, USA) and water (tap water) available ad libitum. Housing in metabolic cages for 24 h were used to determine food consumption. All aspects of animal care and experimentation were performed in accordance with the Guide for the Care and Use of Laboratory Animals published by the US National Institutes of Health (NIH Publication No. 85‐23, revised 1996) and the Italian Laws (D.L.vo 116/92and following additions), which enforces EU86/609 Directive.

P53 genotypes was determined by PCR HotStartTaq kit Qiagen GmbH‐Germany, using the primers: 5′‐AGCGTGGTGGTACCTTATGAG‐3′, 5′‐GGATGGTGGTATACTCAGAGC‐3′ and 5′‐GCTAT CAGGACATAGCGTTGG‐3′; annealing at 72°C and elongating at 58°C.

The L‐368,899, non‐peptidic synthetic compound, selective antagonist of OTR (IC50 = 1.5–15 nM; >40 folds inhibition with respect to the antidiuretic hormone receptor 1a and 2), was purchased from Tocris‐bioscience and dissolved at 20 mg/L concentration in sterilized tap water and provided to the dams directly into the drinking bottle for 19 days, from 48 h after the delivery to the weaning of the offspring at 21 days. The choice to start treatment 48 h after delivery was made to avoid interfering with delivery and the female's recovery soon afterward.

### 2.2 Behavioral assays

Observations in breeding cages were performed in three alternate days, during the midday, enduring for 30 min with white lights on and mice inactive, beginning at day 5 till day 10 postpartum. Test cages were not disturbed except for the observations and for removal of dead puppies and replenishing food and water. Off nest behavior of dams took account of: cannibalism, exploratory activities such as climbing, self‐grooming, laying alone, or drinking/eating; on nest behavior included: pup licking, active nurturing, or passive nursing.^24^

Retrieving behavior was assessed through the nest building test as previously described.^25^ Similarly, inactive caring behavior was monitored by assessing nest destruction events in three independent observation periods of 30 min.

Ultimately, restraint stress was mimicked by placing animals in open‐ended Plexiglas cylindrical restrainers (trailveiner) measuring 6.7 cm in diameter and 22.3 cm in length. Restraint lasted for 15 min/day, at which point animals underwent bleeding to assess cortisol concentration in the serum (next paragraph). Mice were subsequently returned to their home cage.

### 2.3 Glycemia and cortisol quantification, immunohistochemistry

Blood glucose level was measured after overnight starvation by using Accu‐chek glucose meter (Roche). Cortisol concentration in the serum was assessed early in the afternoon upon mouse bleeding. Cortisol concentration in the serum was measured by using the Mouse/Rat Cortisol ELISA Kit SE120082 (Sigma‐Aldrich, USA) following manufacturer instruction.

Upon necroscopy, histological analysis of the organs was performed by formalin‐fixation and paraffin‐embedding procedures followed by the staining of the slides with hematoxylin/eosin or Giemsa dies and immunohistochemical procedures. Anti‐Ki‐67 and ‐AIF‐1 antibodies (Invitrogen ThermoFisher Scientific USA) were used 1:200 diluted.

### 2.4 Bone marrow cell (BMC) analysis and transplantation

BMC were prepared by flushing mouse femurs and tibias with phosphate buffered saline. BMC suspension was treated with BD Pharm Lyse buffer (BD Biosciences, USA) to remove red cells and then washed and resuspended for flow cytometry analysis^26^ or transplantation.

Cells were stained for FACS at 4°C for 30 min with anti‐Gr1, Mac1, CD3e, B220, Ter119, Sca‐1, ‐c‐Kit, ‐Flk2, and ‐CD34 antibodies. After staining, washed cells were acquired using a Beckman Coulter flow cytometer and data were analyzed using FlowJo software.

For transplantation experiments, C57Bl/6 recipient mice were lethally irradiated and transplanted with 2∙10^6^ total BMC from F1 p53−/− CTRL or OTRI donor mice.

### 2.5 Statistics

Unless otherwise specified, data are presented as mean ± standard deviation. Statistical analysis was performed using Graph Pad Prism 9 software. Two‐sample t‐test was used for assessing group effect. Kaplan–Meier estimator was used for the analysis of survival data considering as time and event, the age of the death of the mouse respectively. Gehan‐Breslow‐Wilconox test or longrank test have been used to compare survival data.

## 3 RESULTS

### 3.1 OTR inhibition in p53 mutant mice decreases maternal care and increases maternal cannibalism

**FIGURE 1:**
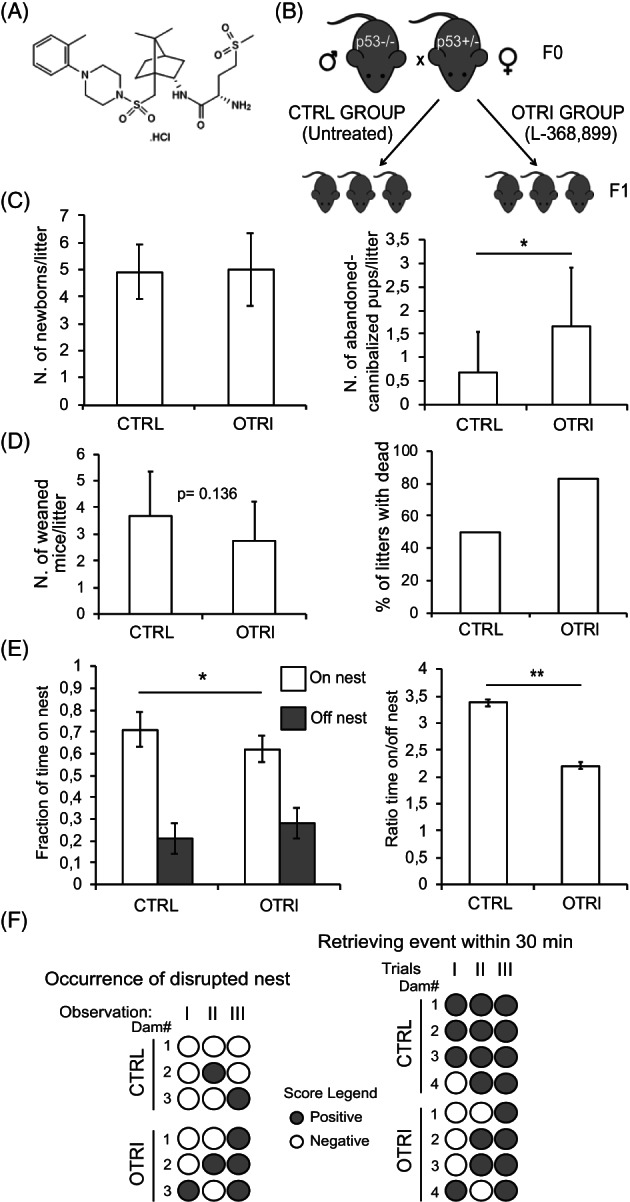
Effect of OTR inhibition on maternal behavior and fertility. (A), molecular structure of the L‐368,899 OTR inhibitor (Tocris bioscience). (B) Scheme of the matings, indicating the two experimental groups, treated (OTRI) or not (CTRL) with L‐368,899 and the two derived progeny groups. (C), average numerosity of litters at birth (left panel) and average number of cannibalized or abandoned puppies per litter from the birth to the weaning (right panel) in the two groups (*n* = 12 litters; *p < .05). (D), average number of weaned mice per litter (*n* = 12 litters; *p* = .136 for the effect of the treatment—left panel) and right panel, percentage of litters with at least one dead pup in the two groups (*n* = 12). (E), average fraction (left panel) of time spent in the nest (white bars) or out of the nest (gray bars) by the dams of the CTRL or OTRI groups (*n* = 8 dams for two replicated observations; *p < .05) and resulting ratio of time (right panel) spent on or off the nest (**p ± <.01). (F), scores of the tests investigating the occurrence of disrupted nest (left panel, *n* = 3 dams observed in triplicates, *p* = .051) and of recomposed nest within 30 min after dismantling it (right panel, *n* = 4 dams observed in triplicates, *p < .05) in the two groups as indicated

In order to alter the mother‐newborn relationship we provided the synthetic OTR antagonist L‐368,899 to dams, from 48 h after the delivery to the end of the weaning (day 21) (Figure 1A).^27^ L‐368,899 was dissolved in the drinking waters, available ad libitum, at a concentration of 20 mg/L, corresponding to a daily intake of the drug of around 3 mg/kg. Sixteen couples (F0 breeders), generated by crossing one p53−/− male and one p53+/− female in the C57Bl/6J mouse strain background were mated. Upon mating, males were removed and females were randomly assigned to two groups (*n* = 8/group). After the delivery, control group (CTRL) received drinking water alone, while the other group (OTRI) received drinking water containing the OT inhibitor L‐368,899 (Figure 1B).

No effects on general health, such as loss of weight, lethargy, altered food or drink consumption, were observed in the treated females. The number of newborns alive at 48 h after birth was the same in both groups but the number of abandoned or cannibalized puppies at 1 week after delivery significantly increased in the OTRI group (Figure 1C). At weaning no significant differences of fertility, that is, number of adult individuals generated by a female, resulted comparing the two groups although the percentage of litters counting dead events was higher if dams were taking the OT inhibitor (Figure 1D).

The effect of OT inhibitor on maternal care was investigated by measuring the time spent by the mother on the litter in the nest and the occurrence of disrupted nest and retrieving performances. Results revealed that dams of the OTRI group spent significantly more time far from the puppies and took less care of the nest (Figure 1E,F).

### 3.2 P53−/− offspring of OTR inhibited dams gained weight normally and showed higher cortisol levels

**FIGURE 2:**
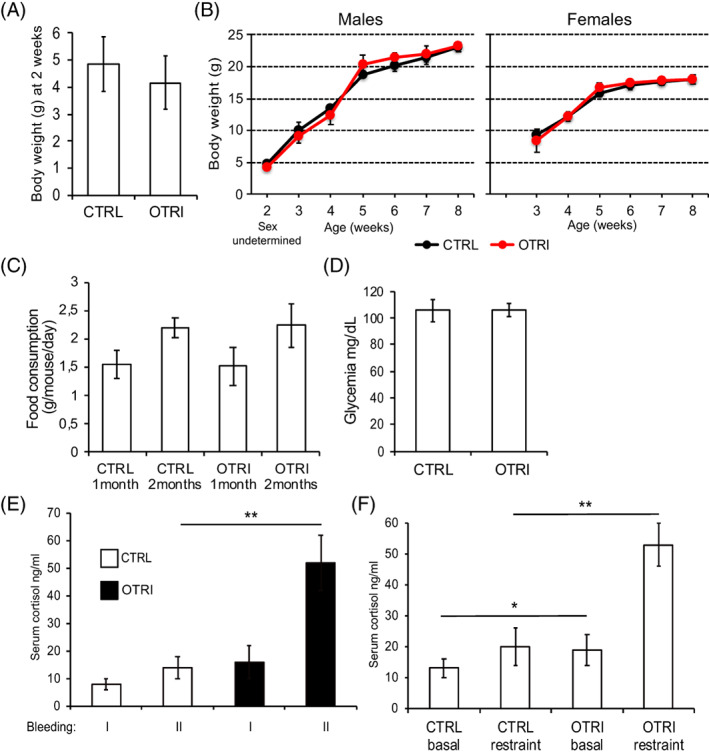
Effect of OTR inhibition on progeny growth and serum cortisol concentration. (A), average body weights of 2 weeks old puppies of the two groups from 4 litters each. (B), body weight of males (left panel; including unclear sex individuals at time 2 weeks) and females (right panel) mice of OTRI (red curves) and CTRL (black curves) groups (*n* = 9 mice); group effect at weaning (time point 3 weeks) for both sex = 0.054. (C), average daily food consumption per mouse at 1 and 2 months of age for the two groups. (D), basal glycemia in 3 months old p53−/− mice in the two groups (*n* = 4). (E), average serum cortisol levels in basal conditions (in two consecutive bleedings). (F), average serum cortisol levels before and after restraint challenge (F) in 3 months old p53−/− male mice (*n* = 4), (*p < .05 and **p < .01 for the effects of groups)

Regardless the reduced maternal behavior of the OTRI dams, their puppies appeared healthy, with regular sign of milk in the stomach at visual inspection. Body weights of un‐weaned offspring appeared slightly reduced, though not statistically significant as sample size was minimal at that stage to avoid interfering (Figure 2A). However, the body weight remained comparable among the offspring of the OTRI group as compared to the CTRL group in the weeks after weaning (Figure 2B). In this respect, mouse body weight was comparable to our historical data on the internal p53 mutant mice colony and to the weight gain of C57Bl/6 strain^28^ regardless the genotype (p53+/− or p53−/−). Further, food consumption and glycemia was also unaffected by the treatment (Figure 2C,D). Interestingly, the frequency of segregation of the p53 null allele in the F1 offspring mice resulted 27% p53−/− in the control and 22% in the OTRI group. These percentages are lower than the expected value resulting from crossing p53−/− males with p53+/− females, as already observed in our p53 colony and by others particularly in females.^29^

To investigate the activity of HPA axis, the levels of cortisol was determined in the serum of 3 months aged p53−/− offspring in basal conditions and after restraint a treatment that stimulates HPA and corticosteroids stress hormones.^30^ As shown in Figure 2E–F, cortisol was significantly higher, particularly upon stress, in the progeny of mothers treated with the OTR inhibitor.

### 3.3 P53−/− offspring of OTR inhibited dams survived less and showed higher incidence of thymic lymphoma

**FIGURE 3:**
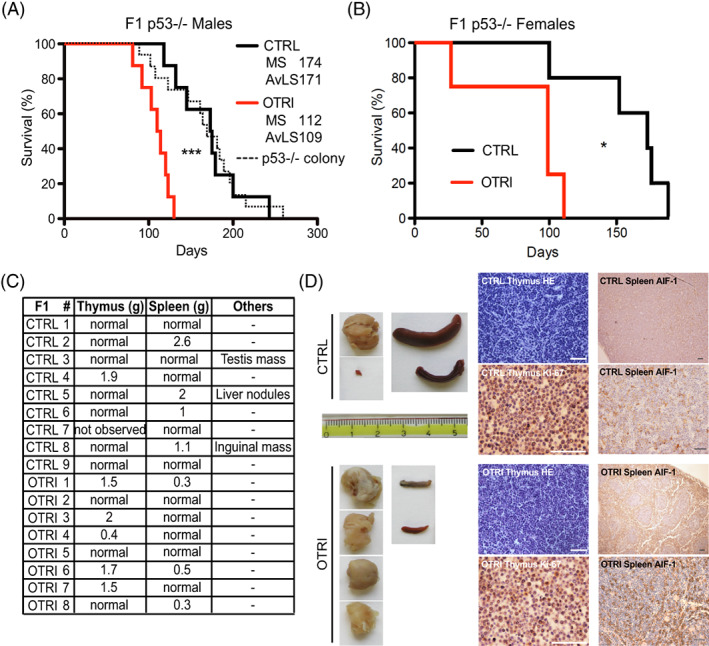
Effect of OTR inhibition on progeny survival and tumor spectrum. Kaplan–Meier survival curves of p53−/− males (A, reporting the estimates of median survival—MS and average life span—AvMS, ***Gehan‐Breslow‐Wilconox test *p* = .0009 or longrank Mantel‐Cox test *p* = .0003) and females (B, *Gehan‐Breslow‐Wilconox Test *p* = .015) of the OTRI (red lines) or CTRL (black lines); The dashed thick black curve in the panel A indicate the survival data of a parallel cohort of p53−/− male mice belonging to the breeding colony as further external control. C, thymus and spleen weights and tumor mass description from the necroscopic reports of OTRI and CTRL p53−/− male mice spontaneously dead during the survival experiments. D, morphology of the largest thymus masses and spleens collected from the mice in the two groups (left panel). Representative immunohistochemical images (right panel) of thymus (HE and anti‐Ki‐67 antibody) and spleen (HE and anti‐AIF‐1 antibody), from the OTRI and CTRL p53−/− mice

Twelve p53−/− F1 (8 males and 4 females) mice from the OTRI group and 13 p53−/− F1 (8 males and 5 females) mice from the CTRL group were maintained undisturbed to determine their survivals. The CTRL mice had a median survival of 174 days and an average life‐span of 171 days as determined by the Kaplan–Meier method (in agreement with former data from our colonies^31^ and published reports^29^). Notably, the median survival and the average life‐span of the OTRI males were 112 and 109 days respectively (Figure 3A). A similar decrease in survival was also observed in females of the OTRI p53−/− group, although their number was limited (Figure 3B). This suggest that the p53−/− progeny of dams treated with the OT inhibitor have shorter average lifespan as compared to the progeny from untreated dams.

After necropsy, the OTRI F1 p53−/− males showed increased incidence of thymoma and rare splenomegaly but no evident tumor masses in soft tissues with respect to the CTRL group (Figure 3C). The histological analysis confirmed thymic lymphomas were the reason of accelerated and prevalent thymomas in OTRI mice (Figure 3D). Coherently, lymphomas are the main cause of death of p53−/− mice.^29^ We did not find any difference in proliferative (Ki‐67, Figure 3D) or cell death (cleaved caspase 3, not shown) markers in OTRI versus CTRL thymus with comparable lesion sizes. However, the spleens from the OTRI group showed a significantly higher expression of the allograft inflammatory factor 1 (AIF‐1), which identifies activated macrophages (Figure 3D). Therefore, our data suggest that reduced parental care in early life statistically increases the risk of lymphoma development in newborn p53−/− mice later on.

### 3.4 BMC from p53−/− mice raised by dams treated with the OTR inhibitor showed increased tumorigenic potential

Several factors, including systemic stress, hormone‐dependent immunomodulation or metabolic adaptation, may contribute to the different survival and tumorigenesis observed in the p53−/− mice subjected to reduced maternal care. To determine whether cell autonomous mechanisms are involved in the increased tumor development observed in the OTRI group, we studied the growth of bone marrow cells obtained from OTRI treated mice transplanted into recipient mice.

**FIGURE 4:**
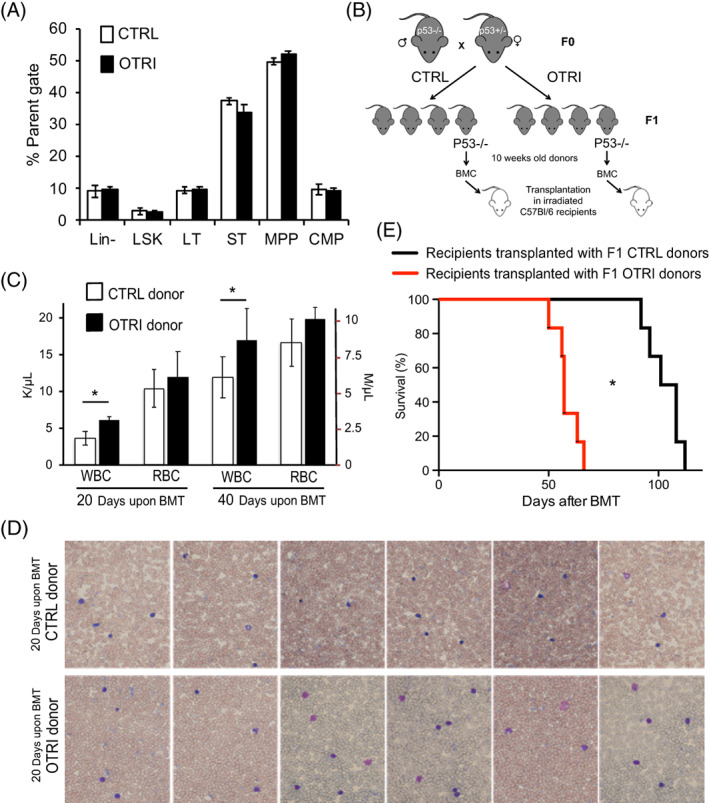
Composition and tumorigenic potential upon transplantation of OTRI and CTRL BMC. (A), flow cytometric analysis of bone marrow subpopulations in OTRI and CTRL p53−/− mice; lineage markers negative (Lin^−^), Lin^−^Sca‐1^+^c‐Kit^+^ (LSK), Lin^−^c‐Kit^+^Sca‐1^+^Flk2^−^CD34^−^ long‐term (LT) and Lin^−^c‐Kit^+^Sca‐1^+^Flk2^−^CD34^+^ short‐term (ST) hemopoietic stem cells, Lin^−^c‐Kit^+^Sca‐1^+^Flk2^+^ multipotent (MPP) and Lin^−^c‐Kit^+^Sca‐1^−^CD34^+^ common myeloid (CMP) progenitors. (B), scheme of the bone marrow transplantation (BMT) experiment of OTRI and CTRL p53−/− groups of mice. (C) Average white (WBC) and red (RBC) blood cell counts, in peripheral blood of recipient mice at 20 or 40 days after BMC transplantation with OTRI (black bars) and CTRL (white bars) donor mice (*n* = 6, *p < .05). (D) Representative images of Giemsa stain of peripheral blood smears collected at 20 days from the 6 recipient mice transplanted with BMC from the OTRI and CTRL. (E), Kaplan–Meier survival curves of recipient mice after bone marrow transplantation with BMC from the OTRI (red lines) or CTRL (black lines) mice (*n* = 6, *Gehan‐Breslow‐Wilconox Test *p* = .014)

The composition of bone marrow hematopoietic compartment appeared comparable between the F1 p53−/− CTRL and OTRI 10 weeks old mice as revealed by FACS analysis using antibodies against cell surface markers (Figure 4A). We transplanted 2∙10^6^ total BMC from F1 p53−/− CTRL or OTRI donor mice into irradiated C57Bl/6 recipient mice (Figure 4B). Hemocytometry analysis of recipient mice revealed a statistically significant higher WBC counts at both 20 and 40 days after transplantation (Figure 4C). Interestingly, recipient mice transplanted with BMC from OTRI mice displayed a sign of lymphoblastic leukemia (Figure 4D). Consistently, mice transplanted with OTRI BMC displayed a significantly shorter survival than mice transplanted with CTRL BMC (Figure 4E).

These findings indicate that a defective maternal care in p53−/− mice progeny induces a stressed phenotype that ultimately promotes intrinsic cellular mechanisms of tumoral transformation.

### 3.5 Distress and tumorigenic effects induced by reduced maternal care due the OTR inhibition partially transmits to the second generation

**FIGURE 5:**
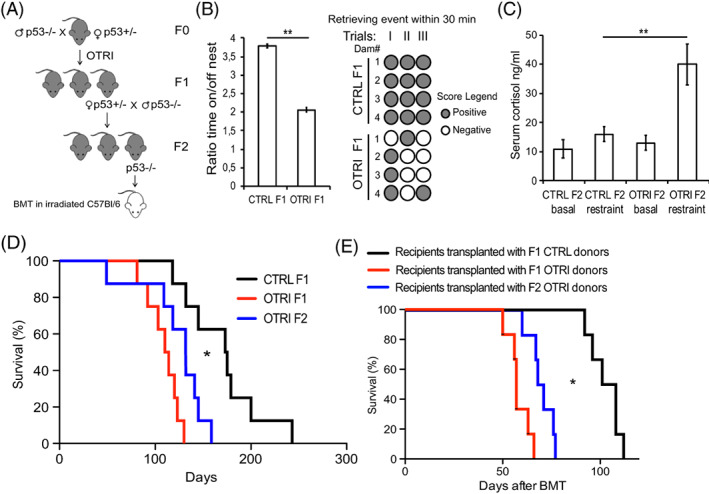
Phenotypic analysis of second generation of OTRI treated dams. (A), scheme of the genealogy of OTRI and CTRL groups with the different generations investigated (F0, breeders treated with the OTR inhibitor; F1, first generation and F2, second generation) and the transplantation experiment (BMT) from donors of the F2. (B), ratio of time spent on or off the nest (left panel) by the OTRI and CTRL F1 p53+/− groups of dams (*n* = 4; **p < .01); occurrence of recomposed nest (right panel) within 30 min after the dismantling in the OTRI and CTRL F1 p53+/− dams (*n* = 4 dams/group observed in triplicates, **p < .01). (C), average serum cortisol levels before and after restraint challenge in 3 months old F2 mice (*n* = 4 **p < .01) originated from the OTRI and CTRL groups. (D), survival curves of F2 p53−/− males (*n* = 8, blue line) of the OTRI group and of the F1 OTRI (red line) and CTRL (black line) p53−/− males (*Gehan‐Breslow‐Wilconox Test *p* = .0203 or longrank Mantel‐Cox test *p* = .008 for the comparison OTRI F2 vs. CTRL F1; for the comparison of OTRI F2 vs. OTRI F1 Gehan‐Breslow‐Wilconox Test *p* = .07). (E), survival curves of recipient mice after bone marrow transplantation with BMC from the F2 OTRI (blue line, *n* = 6) or F1 OTRI (red lines) or CTRL (black lines) p53−/− male mice (*Gehan‐Breslow‐Wilconox Test *p* = .013 for the comparison F2 OTRI donors vs. F1 CTRL donors; for the comparison OTRI F2 donors vs. OTRI F1 donors Gehan‐Breslow‐Wilconox Test *p* = ns)

The maternal bond mediates an additional form of transgenerational inheritance of phenotype as stressed progeny will provide poor parental care and stressed phenotype to the succeeding generation as well.^32^ Thus, we investigated whether the consequential effect of OT inhibitor treatment on maternal behavior and subsequent enhancement of stress in their progeny could be transferred to F1 generation. In order to do that, F2 generation were produced after mating F1 p53+/− females with p53−/− males from the colony (Figure 5A).

We found that the maternal behavior of the F1 dams generated from the OTRI group was impaired as their nurture time and retrieving performances were decreased as compared to the behavior of F1 dams generated from the CTRL group (Figure 5B). Consistently, the serum cortisol levels were significantly higher in the progeny (F2) of OTRI dams as compared to the progeny (F2) of CTRL dams upon restraint, even though the basal levels were comparable. These data suggest that F2 mice display a susceptible HPA axis and a low resilience to stress (Figure 5C).

Strikingly, the survival of the F2 OTRI p53−/− males, though improved with respect to the F1 OTRI group, was still shorter than the F1 CTRL p53−/− males (Figure 5D) with a median survival and average life span of 132 and 123 days respectively with respect to 174 and 171 days observed in the F1 CTRL group.

Finally, transplantation experiments of BMC obtained from F2 OTRI p53−/− mice into C57Bl/6 irradiated recipients revealed increased tumorigenic potential with respect to the CTRL group. In fact, recipient mice transplanted with OTRI F2 p53−/− BMCs showed a reduced survival (median survival = 69.5 days) as compared to recipient mice transplanted CTRL F1 p53−/− BMCs (Figure 5E). Therefore, the distress caused by poisoning oxytocin receptor in dams imprints shorter life span, increased cancer risk and higher tumorigenic capacity of BMCs in later generations.

## 4 DISCUSSION

Early life is a critical time period for all species as somatic and germ cells complete development and unfavorable events such as nutrient,^33^ heat or oxygen^34^ deprivation can have long‐term consequences, possibly inheritable from one generation to the next one.^35^ In humans, adverse conditions during early life have been found to favor several disorders including obesity,^36^ cognitive dysfunctions,^30^ inflammation,^37^ and to increase the incidence of life‐threatening cardiovascular diseases and cancer.^38^ Parental care is essential, especially in mammals, that buffers detrimental experiences to newborns, and ensures the fitness of the progeny.^10^ Therefore, sub‐optimal newborn‐mother, or ‐father,^39^ interaction induces maladaptation^40^ that persists over the life span and has been hypothesized as a population risk factor for severe diseases and mortality.^41^

Although several epidemiological studies pointed out child abuse as a cancer risk factor,^3^ only two studies in humans revealed that poor parental care is associated to increased incidence of different cancer types^4^ and worse breast cancer prognosis.^5^ In animal models, the removal of the dams from the puppies was found to exacerbate breast carcinogenesis upon adulthood administration of 7,12‐dimethylbenzo[*a*]anthracene in BALB/c mice^6^ and in Wistar rats.^7^ Similarly, reduced parental care was shown to accelerate the progression of melanoma and breast cancer xenografts progression in C57BL/6 mice^8^ or Fischer 344 rats.^9^However, postnatal maternal separation does not provide a robust model to study the effect of early life stress on progenies health.^42^ Pharmacological and genetic inhibition of OTR, a hormonal circuit involved in essential maternal behavior is widely used to study the consequences of impaired maternal care on offspring's health.^21^

In order to study the effect of impaired maternal care specifically on spontaneous tumorigenesis, we have used p53 null mice model. P53 null mice bestow an attractive tumorigenesis model as tumor development in these mice is rapid and spontaneous.

Here, we have used a synthetic OTR antagonist, L‐368,899, that have been previously used to inhibit oxytocin system and shown to alter social behavior and attraction in pair‐bonding,^22^ However, the effect of this OTR antagonist on maternal bond is not well studied. As expected, inhibition of OTR in p53 null mice using synthetic OTR antagonist decreases the maternal care and increases the maternal cannibalism. The treatment does not affect the body weight, food consumption or fertility of the treated female. However, they showed less care towards their puppies and had reduced nest‐building behavior. Although, this may have caused a slight decrease in the weight gain in the un‐weaned puppies, however, after weaning these puppies gained and behaved similarly to the puppies of untreated mothers. A deficit in parental care is known to alter the neuroendocrine responses to stress in adulthood by dysregulating the HPA axis,^43^ which is in turn supposed to mediate the tumorigenic effect of psychological stress.^44^ Indeed, the level of stress hormone, cortisol, was found to be higher in the puppies of OTR antagonist treated mothers both under basal and under psychological stress. Besides that, the puppies of the OTR antagonist treated mothers seems to be more prone to thymic lymphoma and have a decreased life‐span as compared to the puppies of untreated mother. Additionally, defective maternal care promotes intrinsic cellular mechanisms of tumoral transformation in p53 mice progeny. In all, suggesting that altered maternal bond during early life represents a detrimental risk factor for tumor development in the p53 null tumor‐prone mouse model. Notably, the puppies of the treated mother display a defective parental care phenotype as well, thereby leading to transgenerational inheritance of increased cancer development risk. Our findings substantiated the transgenerational effects of altered maternal care on cancer risk suggesting an evolutionary role of parental‐offspring interaction.^45^ In this regard, the OT circuit has been suggested to play a critical role for transmission of maternal misbehavior through generations.^46^

At mechanistic level, several systemic or cellular processes may be involved in the adulthood tumorigenic effect of maternal OTR inhibition. Maternal separation in rats has been shown to reduce natural killer cell activity and thereby suggested to suppress tumor immunity.^9^ In our model, defective parental care affected tumor size and spectrum rather than cellular characteristic within tumor. In fact, when we analyzed thymomas in the OTRI and CTRL groups, we noticed a comparable proliferative potential, in spite of the higher tumor incidence in the OTRI group. Therefore, it is possible that *i*) systemic phenotype might account for the enhanced tumor susceptibility scored in the OTRI animal group, or alternatively, *ii*) maternal OTR inhibition might increase tumor susceptibility by regulating the number and features of tumor initiating cells in the progeny. In agreement with the first scenario (*i*), we scored enhanced macrophage activity in OTRI mice (Figure 3D) which may be related to the altered levels of corticosteroids. Interestingly, maternal separation approaches in mice were also found to remodel transcription in specific neuronal cells^47^ and, therefore, we hypothesized that cells from other tissues adapt transcription upon early life stress acquiring an oncogenic phenotype. In this respect, the observation that BMCs of following generations retain an increased tumorigenic potential highlights the involvement of stable intrinsic cellular alterations. These alterations may be selected in tumorigenic initiating cells growing in a stressed organismal environment due to the hormonal unbalance. Alternatively, as p53 null background is particularly sensitive to mutagenic factors, mutation rate may be increased in early‐life stressed mice leading to accelerated accumulation of critical oncogenic mutations in stem cells.

In conclusion, although we are aware that our study displays some limitations, namely the study of maternal behavior in captivity, the lack of functional p53 that jeopardizes its contribution to the stress response,^48^ the metabolic effects of antagonizing OT and the possibility of an off‐targets activity of the drug acting on unpredictable mechanisms of tumorigenesis, we strongly believe that the findings presented here shed light on parental care role in spontaneous tumor development in the progeny. Further studies are warranted to unravel the molecular reasons of increased tumorigenesis in mice that received poor maternal care.

## AUTHOR CONTRIBUTIONS

**Massimo Stendardo:** Investigation (lead); methodology (lead). **Chiara Renzi:** Conceptualization (equal); data curation (equal); formal analysis (equal); methodology (equal). **Rani Pallavi:** Investigation (equal); methodology (equal). **Niccolò Roda:** Data curation (equal); formal analysis (equal); writing – original draft (equal). **Valentina Gambino:** Data curation (equal); formal analysis (equal); writing – original draft (equal). **Francesca Casciaro:** Data curation (equal); formal analysis (equal). **Giuseppe Persico:** Data curation (equal); formal analysis (equal). **Marco Giorgio:** Conceptualization (lead); data curation (lead); formal analysis (lead); funding acquisition (lead); investigation (equal); methodology (equal); supervision (lead); writing – original draft (lead).

## CONFLICT OF INTEREST

The authors have stated explicitly that there are no conflicts of interest in connection with this article.

## ETHICS STATEMENT

The experiments described in our study were conducted according to accepted standards of humane animal care and were consistent with federal guidelines and Directive 2010/63/EU of the European Union. Experimentation involving the p53 KO mice was approved, prior to commencing this study, by the institutional review board and notified to the Ministry of health (project # 22/2010‐12).

## Data Availability

The data will be made available upon mail request to the authors.
